# Ockham’s razor for the MET-driven invasive growth linking idiopathic pulmonary fibrosis and cancer

**DOI:** 10.1186/s12967-016-1008-4

**Published:** 2016-09-02

**Authors:** Giulia M. Stella, Alessandra Gentile, Alice Baderacchi, Federica Meloni, Melissa Milan, Silvia Benvenuti

**Affiliations:** 1 Pneumology Unit, Cardiothoracic and Vascular Department, IRCCS Policlinico San Matteo Foundation and University of Pavia Medical School, Piazzale Golgi 19, 27100 Pavia, Italy; 2 Investigational Clinical Oncology (INCO), IRCCS Candiolo Cancer Institute-FPO, Candiolo, 20060 Turin, Italy; 3 Experimental Clinical Molecular Oncology (ECMO), IRCCS Candiolo Cancer Institute-FPO, Candiolo, 20060 Turin, Italy

**Keywords:** Cancer, Idiopathic pulmonary fibrosis, Invasive growth, Epithelial-to-mesenchymal transition, Precision medicine

## Abstract

**Background:**

Idiopathic pulmonary fibrosis (IPF) identifies a specific lung disorder characterized by chronic, progressive fibrosing interstitial pneumonia of unknown etiology, which lacks effective treatment. According to the current pathogenic perspective, the aberrant proliferative events in IPF resemble those occurring during malignant transformation.

**Main body:**

Receptor tyrosine kinases (RTK) are known to be key players in cancer onset and progression. It has been demonstrated that RTK expression is sometimes also altered and even *druggable* in IPF. One example of an RTK—the MET proto-oncogene—is a key regulator of invasive growth. This physiological genetic program supports embryonic development and post-natal organ regeneration, as well as cooperating in the evolution of cancer metastasis when aberrantly activated. Growing evidence sustains that MET activation may collaborate in maintaining tissue plasticity and the regenerative potential that characterizes IPF.

**Conclusion:**

The present work aims to elucidate—by applying the logic of simplicity—the bio-molecular mechanisms involved in MET activation in IPF. This clarification is crucial to accurately design MET blockade strategies within a fully personalized approach to IPF.

## Background

Idiopathic pulmonary fibrosis (IPF) is characterized by progressive scarring of the lungs ultimately leading to severe respiratory failure and death [1]. The median survival of patients is only 3 years following diagnosis [2], similar or worse than that of several oncologic diseases. Despite the fact that recently significant progress has been made in identifying of the bio-molecular mechanisms related to the development of IPF, a better understanding of disease pathogenesis is needed to identify more effective therapies and to improve patients’ outcome. According to the current pathogenic perspective, the aberrant proliferative events in IPF resemble that occuring during malignant transformation. Growing evidence supports the cancer-like molecular nature of IPF and this intriguing hypothesis is now also being exploited for therapeutic purposes [3]. The discovery of pathogenic links between the two diseases may have practical consequences in encouraging the use of cancer drugs for treating IPF. Receptor tyrosine kinases (RTK) are known to be key players in cancer onset and progression; it has also been demonstrated that the expression of some RTK family members is also altered and even *druggable* in IPF [4, 5]. The multi-kinase inhibitor—nintedanib—was initially developed for cancer, and has now been approved for the treatment of IPF thanks to the observation that targeted receptors are also abnormally activated in IPF [6]. The MET proto-oncogene is a RTK that is a key regulator of invasive growth [7], which is the biological program that orchestrates dynamic changes in tissues leading to cell proliferation, survival and migration across the extracellular matrix (ECM) and which can be inappropriately overexpressed in cancer spreading and metastatization. On the other hand, the MET-induced invasive growth is now emerging as potential target in IPF, although some issues require better understanding and clarification. Thus, this review aims to analyze the multiple facets of MET activation in cancer and IPF, under a context-specific perspective to the fibrotic disease.

### The empirical evidence: MET structure and signaling

**Fig. 1:**
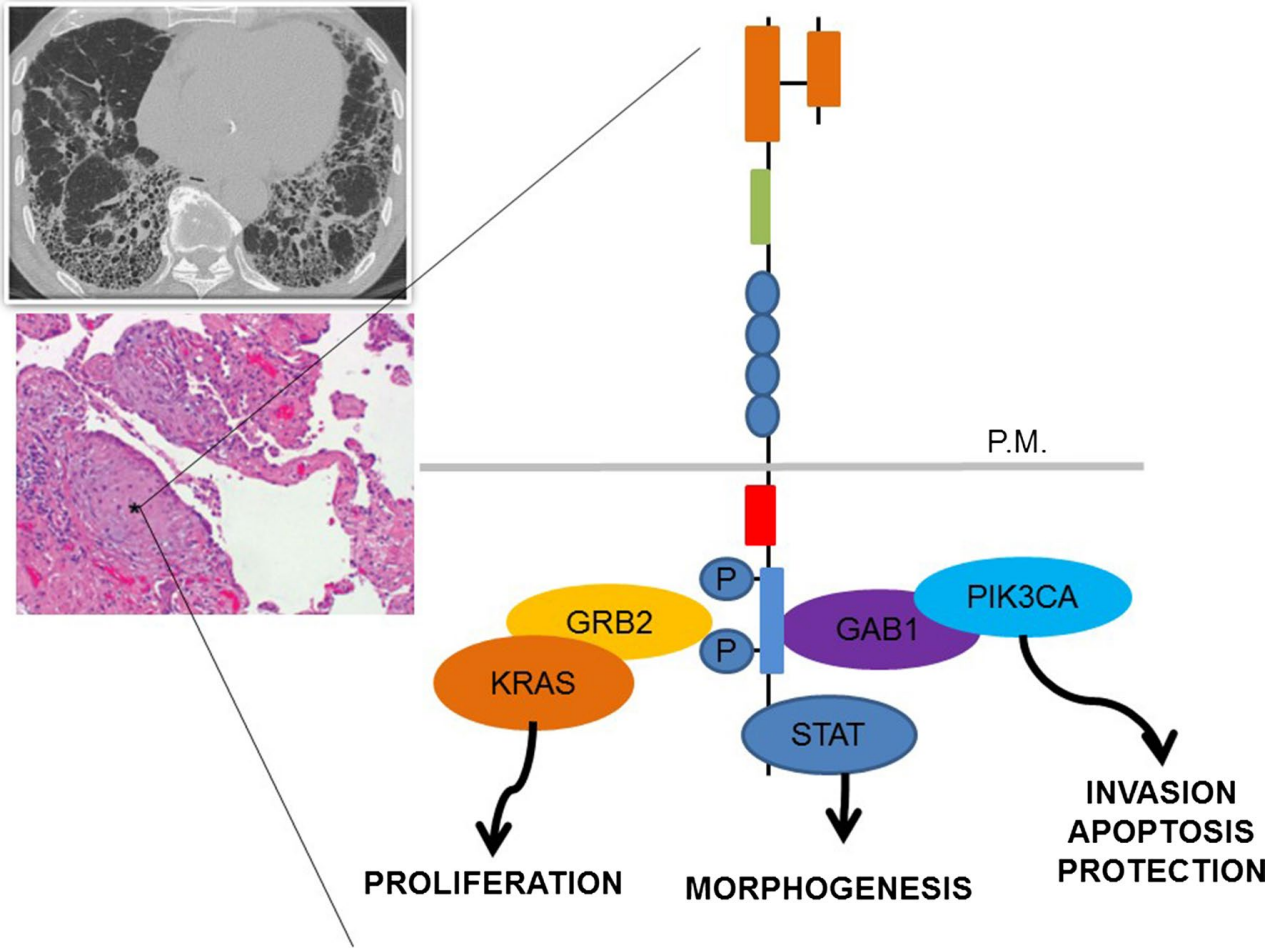
MET signaling pathway in IPF. Enhanced MET activation controls genetic programs leading to cell growth, invasiveness and protection from apoptosis. For both the biological and therapeutic implications of MET activation in myofibroblasts in FF, its KRAS-driven pro-proliferating activity can be separated from the PI3CA-related pro-invasive role. The activity of branching morphogenesis depends on STAT family members, mainly STAT3 (Giordano et al. [100]). STAT3 is known to contribute to lung damage in IPF onset and progression (Pedroza et al. FASEB J 2016; 30(1):129–4)

The MET proto-oncogene is a key regulator of the genetic program known as invasive growth. The *MET* gene, located on chromosome 7q31, encodes for the TK receptor for ‘Scatter Factor’ or Hepatocyte Growth Factor (HGF), which detects adverse micro-environmental conditions and drives cell invasion and metastasis through the transcriptional activation of the *invasive growth signature*, a genetic program also defined as the epithelial-mesenchymal transition (EMT) [8]. The latter includes cell–cell dissociation and scattering, migration, cellular proliferation, resistance to *anoikis* and angiogenesis. MET is now a prominent target in cancer therapy, with several compounds in active clinical development (Table 1). Different strategies have been pursued to inhibit MET, each focusing on one of the sequential steps that regulate MET activation. Scatter factors (HGF and Macrophage-stimulating Protein-MSP) belong to the plasminogen family of proteins, which is defined by the presence of at least one characteristic domain known as the kringle domain (an 80 amino-acid double-looped structure formed by three internal disulphide bridges); a serine-protease domain and an activation segment that is located between the kringle and the protease domains. HGF and MSP are unique scatter factors because they lack proteolytic activity; they are secreted as single-chain biologically inert glycoprotein precursors and are converted into their bioactive form in the extracellular environment by specific proteases, which break the bond between two positively charged aminoacids (Arg494–Val495). The mature factors are heterodimers consisting of an α-chain and a β-chain held together by a disulphide bond. The MET receptor for HGF and the RON receptor for MSP are single-pass, disulphide-linked α/β heterodimers that are formed by proteolytic processing of a common precursor in the post-Golgi compartment. In both receptors (which share 63 % overall homology), the α-chains are completely extracellular, whereas the β-chains are transmembrane subunits that contain tyrosine kinase activity. The extracellular region of these receptors displays structural analogies with the extracellular domains of semaphorins (a large family of secreted and membrane-bound proteins) and their receptors plexins. The extracellular region contains the sema domain: a conserved sequence of about 500 amino acids comprising an eight-cysteine peptide module that is conventionally termed MRS (MET-related sequence), together with three glycine-proline rich (G-P) repeats. The intracellular domains include tyrosine kinase catalytic sites that are flanked by distinctive juxtamembrane and carboxy-terminal sequences. Phosphorylation of MET on residues Tyr 1234 and Tyr 1235 within the catalytic sites results in positive modulation of enzyme activity, whereas phosphorylation of a serine residue in the juxtamembrane domain downregulates the kinase. After activation, MET elicits intramolecular phosphorylation of the other two critical tyrosine residues at the carboxy-terminal domains (Tyr 1349 and Tyr 1356), at the C-terminal of the α-chain: these two sites, together with the surrounding aminoacids, constitute the so-called “multifunctional docking site”, a motif which, when activated after phosphorylation, induces a series of biological processes that ultimately lead to invasive growth. The specificity of this unique response is determined by qualitative activation of specific pathways that are responsible for the oncogenic and migratory effects of MET [for a full review see 9–11], (Fig. 1). Moreover activation of the MET receptor is known to promote a cancer-associated thrombo-hemorrhagic syndrome that is mediated by transcriptional up-regulation of the pro-coagulation factors plasminogen activator inhibitor type-1 and cyclooxygenase-2 [12]. In human tumors, MET activation can be induced through different mechanisms, namely: (i) MET over-expression, related to: *MET* gene amplification; enhanced *MET* transcription; induction by other oncogenes such as *RAS*, *RET*; hypoxia-activated transcription; (ii) structural alteration, such as: point mutations which cause increased kinase activity; oncogene rearrangement (such as chromosomal translocation responsible for the Trp-MET fusion protein); abnormal post-translational processing resulting in a constitutively active molecule exposed on the cell membrane; impaired down-regulation generally due to mutations that prevent binding of the Cbl ubiquitin ligase which is responsible for MET ubiquitination and endocytosis thus leading to increased receptor expression at cell surface and enhanced signal transduction. In addition, naturally truncated and active MET receptors have been detected in malignant human musculoskeletal tumors; (iii) HGF-independent autocrine-paracrine activation. In these contexts, paracrine activation—typical of physiological conditions—can become pathological in the presence of abnormal HGF production by mesenchymal cells. Autocrine activation occurs when tumor cells aberrantly express both HGF and its receptor, as observed in rhabdomyosarcomas, gliomas, carcinomas of thyroid, breast and lung cancers, (iv) HGF-independent mechanisms. Moreover MET phosphorylation can also occur through transactivation by other membrane receptors, including adhesive receptors such as CD44, integrins, signal transducing receptors (RON, EGFR family members, FAS, plexin B9) [for a detailed review see 11, 13]. In addition, it has been demonstrated that MET activation can be mediated by an interaction between MET and microbes, including *H. Pylori*, associated with gastro-esophageal reflux disease which, in turn, is known to be implicated in the development of IPF, giving rise to recurrent lung insult [14, 15]. Notably, MET is expressed in stem and committed progenitor cells and the MET-driven invasive growth is usurped by cancer stem cells (CSC) [7]. As for normal tissues, tumors are structured according to a hierarchy, which includes two main components; thus tumors are composed of tumor-initiating cells (TICs), known as CSCs, which are the small fraction of cells within a tumor mass featuring self-renewal potential, capability of continuous proliferation and the ability to initiate tumor formation when transplanted. TICs also sustain tumor regeneration, growth and dissemination and can be considered the key target of cancer inhibition. Conversely, the vast majority of the tumor is constituted of cells with limited proliferative properties, which tend to aberrantly differentiate and ultimately die [16, 17]. Rapid progress has been made regarding CSC expression of metabolic regulation markers, growth factors, and transcription factors [18, 19]. A greater knowledge of the biological mechanisms responsible for maintaining the stem phenotype is required to understand more fully how the stem compartment sustains tumor persistence, and leads to recurrence after tumor dormancy and failed therapies. In this way, the clinical management and therapeutic options for cancer can be improved. The MET oncogene is crucial to sustain CSC and self-maintenance of tumors. A number of studies have documented the involvement of MET in TIC plasticity [20–23] in several cancer types and in inducing CSC chemo- and radio-resistance [24–26]. In conclusion the MET-driven invasive growth is necessary for efficient cancer spreading as well as stemness properties. Due to its overlapping biological functions MET activation influences IPF development and progression. We and others have already reported that both myofibroblasts and epithelial cells of fibroblast foci in IPF harbor MET in its activated form [27, 28]. Although the anti-fibrotic effect of the MET-ligand HGF, is well known [11, 29], deregulation of the MET signaling cascade is clearly implicated in the development of IPF but its exact role remains to be clarified.

**Table 1:** Details on anti-MET agents already available in the clinical scenario for anticancer therapy

Drug	Target	Cancer type	References
Anti-MET monoclonal antibodies			
SAIT301	MET	Advanced MET positive solid tumors	[30]^a^
ARGX-111	MET	MET protein overexpressing advanced cancer	[31]^a^
MetMab (ornatuzumab)	MET	Advanced or metastatic solid tumors	[32–37]^a^
JNJ-61186372	MET-EGFR (bispecific ab)	Advanced NSCLCs	[38–40]^a^
ABT-700	MET	Advanced solid tumors	
MET tyrosine kinase inhibitors			
PF-02341066 (crizotinib)	MEK, ALK, ROS 1 (triple inhibitor)	Advanced NSCLCs, gastric cancers, metastatic urothelial cancers,anaplastic large cell lymphoma, colorectal cancers, advanced relapsed/refractory solid tumors, primary CNS tumors	[41–43]^a^
XL-184 (cabozantinib)	MET, VEGFR2 (dual inhibitor)	NSCLCs with brain metastasis, advanced cholangiocarcinoma, metastatic triple negative breast cancers, colorectal cancers, metastatic Merkel cell carcinoma, recurrent endometrial cancers, breast cancers with brain metastasis, metastatic renal cell carcinoma	[44–54]^a^
AZD6094 (Volitinib)	MET	Gastric adenocarcinoma, papillary renal cell carcinoma	[55–58]^a^
GSK1363089 (Foretinib)	MET, VEGFR2 (dual inhibitor)	Papillary renal cell carcinoma, medulloblastoma, metastatic gastric cancers, hepatocellular carcinoma	[59–67]^a^
AMG337	MET	Advanced gastric and esophageal adenocarcinoma, advanced solid tumors	[68–72]^a^
ARQ-197 (tivantinib)	MET (non ATP-competitive)	Relapsed/refractory multiple myeloma; locally advancer or metastatic colorectal cancers; metastatic triple negative breast cancers; childhood relapsed/refractory solid tumors; recurrent/metastatic head and neck cancers; gastric cancers; metastatic solid tumors; metastatic prostate cancers; metastatic or locally advancer kidney cancers; mesothelioma; small cell lung cancers; hepatocellular carcinoma;	[73–90]^a^
INC280 (capmatinib)	MET	NSCLCs; CRCs; HNSCC; advanced solid tumors, hepatocellular carcinoma; metastatic CRCs; metastatic renal cell carcinoma; recurrent glioblastoma; advanced or metastatic melanoma	[91, 92]^a^
EMD 1204831	MET	Advanced solid tumors; advanced hepatocellular carcinoma	[93]^a^
MGCD265	MEK, ALK (dual inhibitor)	Advanced cancers	[94, 95]^a^
MK8033	MET, RON (ATP-competitive dual inhibitor)	Advanced solid tumors	[96]^a^
PF-04217903	MET (ATP-competitive)	Advanced cancers	[97–99]^a^

^a^For more details see: www.clinicaltrials.gow

### Competing hypotheses: the role of MET in IPF

IPF is a proliferative disorder affecting the lungs, characterized by aberrant deposition of ECM and consequent remodeling associated with the activation of fibroblasts as a response to still unknown injuries. IPF diagnosis is confirmed by histological identification of the usual interstitial pneumonia (UIP) pattern on surgical (and rarer transbronchial) biopsies, together with detection at high resolution (HR) computerized tomography (CT) scan of bibasilar reticular abnormalities (honeycombing pattern) with minimal or absent ground-glass opacities [1, 101, 102]. The key histological feature of IPF is represented by the so-called *fibroblast foci* (*FF*) defined as aggregates of actively proliferating fibroblasts and myofibroblasts. Activated fibroblasts express α-SMA (smooth muscle actin), accounting for to term “myofibroblasts”. In addition they secrete increased levels of ECM-degrading proteases (metalloproteinases MMP2, MMP3, MMP9), facilitating increased ECM turnover and altered ECM deposition; they also secrete growth factors (such as HGF, IGF, NGF, WNT1 and EGF) which can induce proliferative signals within adjacent epithelial cells. Moreover activated fibroblasts behave as modulators of the immune response following tissue injury by secreting cytokines (e.g. IL-1) and chemokines (e.g. MCP1) [103–107]. Activated fibroblasts/myofibroblasts can be found in wound healing processes and sclerosing tissue and as well as in cancers [108]. Through embryogenesis, cells start to move out from developing tissues in order to organize the structure of fetal organs. In a similar fashion, in adult life, during wound healing and tissue repair processes, health cells migrate into the wound to recreate pre-existing tissue patterns [109]. The acquisition of cell motility is required but is not enough to sustain the whole process. Indeed cells need to trigger a number of biological programs, as well as to activate mitotic divisions to repair injured tissues [110]. Thus embryogenesis, tissue repair after wound healing and cancer share similar mechanistic basis, since the same biological activities—cell proliferation, survival and migration, namely the Invasive Growth—are activated in both normal and malignant contexts. During wound healing repair activities as well as in cancer metastatic spreading, several cytokines are secreted in the reactive interstitial compartment. For instance interleukin-1 (IL-1) and 6 (IL-6), tumor necrosis factor alpha (TNF-α) and transforming growth factor beta (TGF-β) are known to induce the transcriptional up-regulation of HGF (in fibroblasts and macrophages) and MET (in epithelial cells) [111, 112]. HGF is also biologically activated, as demonstrated by the overexpression of proteases involved in pro-HGF activation [113, 114]. Moreover, HGF might be activated through an autocrine loop in stromal myofibroblasts. This mechanisms has been well demonstrated during tumor cell invasion [115] but can reasonably be significant in wounds repair as well. Overall, this highly performant HGF assures a proper activation of MET, which is, thus, involved in tissue protective physiological systems. These morphogenetic pathways trigger the EMT by activating biological processes such as cell motility and invasion [116], known as invasive growth program. The aberrant activation of the above described wound healing machinery ultimately characterizes IPF onset and progression. Thus, HGF/MET-driven aberrant morphogenesis plays a crucial role not only in cancer but in IPF, as well. However it should be underlined that its activation and progression in IPF certainly differs from that in cancer, regarding both spatial and temporal characteristics. A proliferating tumor becomes malignant when neoplastic cells move to adjacent environments and settle in tissues and organs that are distant from the original site of growth. In IPF the actively proliferating FFs contrast with neighboring areas of relatively normal parenchyma and move from subpleural regions towards central areas. IPF is overall a lung-specific disease, defined by a centripetal track of disease progression in absence of distant cell scattering. The latter is a key difference with respect to scattering of malignant cells, which essentially means distant and peripheral dissemination. Furthermore IPF is a heterogeneous disease also in the age of lesions, meaning the stage of pathology in different lung parenchymal regions. Thus normal lung tissue is interspersed with interstitial fibrosis, honeycomb cysts and fibroblast foci [1]. On the other hand, it is well known that most tumors tend to become more aggressive in clinical behavior over time, although this time course may be variable. During cancer progression, MET activation generally occurs as a late event, as a consequence to transcriptional up-regulation driven by unfavorable microenvironmental conditions, such as hypoxia or ionizing radiation [7, 117]. Sometimes, rapidly invasive cancers are diagnosed because of appearance of metastatic lesions in absence of a clearly detectable primary mass. Among these highly invasive and malignant tumors, an extremely high mutational frequency of *MET* coding sequence has been reported; *MET* mutations have been biologically associated to the observed transformed phenotype [118]. The above described differences between IPF and cancer strictly reflect the differences of cell lineages. Indeed cancer is, by definition, a disease of genes, which evolves through a dynamic process of clonal expansion and selection in of advantageous somatic driver lesions [119, 120]. Each individual tumour is defined by a unique clonal evolution resulting from an intricate connection between genetic and non-genetic/epigenetic factors, leading to phenotypic and genotypic heterogeneity. Among the diversity in tumor-cell population, the CSC compartment brings about tumor maintenance and progression [121]. MET-driven invasive growth is aberrantly activated in cancer, mainly as a late event, leading to distant dissemination and malignant progression. More recent studies have reported that MET amplified cancer clones are selected under therapeutic pressure in a context of molecularly heterogeneous lesions exposed to targeted therapies or radiotherapy [8, 122–125]. In CSC, both the occurrence of genetic lesions (as amplification) and physiological expression of MET can contribute to tumorigenesis and therapeutic resistance, by sustaining the invasive growth phenotype. On the other hand, myofibroblasts within FF in IPF are characterized by cellular and genetic heterogeneity. Notably—very recently, Jones and colleagues elegantly demonstrated that FFs in IPF identify—quite unexpectedly—morphologically complex 3D-structures, each independent from the others [126]. These findings strongly suggest that IPF onset relies on the aberrant local responses that are activated and lead to multifocal injuries. As a consequence diffuse cellular fate conversion and tissue plasticity are associated to IPF. During organ regeneration, MET physiological activation displays protective functions: epithelial cells located at the wound edges exploit invasive growth to enhance cellular division and repopulation of the injured areas [127–129]. When the damage inappropriately persists, as in IPF, the HGF/MET pair actively contrasts myofibroblasts activation and the consequent associated abnormal deposition of extracellular matrix [130]. Moreover it is well known that semaphorins might activate MET in and HGF-independent manner. As already presented, MET and plexins share high homology at the extracellular sema domain. When MET oligomerizes with plexins, it can be activated by semaphorins, even in the absence of its ligand HGF [131, 132]. Growing evidence sustains that semaphorins—and their ligands plexins—have a role in enhancing immune function and angiogenesis as well as in controlling lung fibrogenic diseases [133–136]. As a consequence, fibrosing settings, as IPF, which co-express both HGF and plexins might feature even hyperactive invasive capacities.

### Applying the razor: MET as an actionable target for IPF

In a complex and heterogeneous setting, which applies to IPF, the principle of *pluralitas non est ponenda sine necessitate* (Ockham’s razor, principle attributed to the 14th century logician William of Ockham) can be applied to correctly understand the role of MET-driven invasive growth at disease onset. IPF resembles cancer in many MET-associated behaviors, such as invasive phenotype and pro-coagulant status. However dynamics of malignant divergent clonal selective pressure and heterogeneity clearly differ from those occurring in IPF and impact on the biological significance of MET activation. The RTK MET is phosphorylated in myofibroblasts in FF: in a context-specific regulation of its expression, MET might become a functional marker of IPF and an actionable target. The cytogenetic heterogeneity, that is a hallmark of FF, can be exploited for therapeutic purposes and already commercially available MET-inhibitors can be tested to interfere with IPF progression. MET-mediated events in IPF rely on qualitative differences among physiological signals, whereas no driver genetic lesions, causally implicated in the disease can be clearly demonstrated. Thus MET blockage falls among those therapeutic strategies aimed to impair the “aberrant recapitulation of developmental programs” as Selma and coll. already defined IPF [137]. A dynamic crosstalk between MET and developmental signaling pathways which are known to be activated in IPF is well documented. Among them the most relevant are those driven by Wnt/β-catenin and TGFβ cascades [116, 136]. The evolutionary conserved Wnt signaling canonical pathway is known to be a key player in maintaining tissue homeostasis, cell proliferation and differentiation, and in regulating cell renewal and differentiation. Wnt signaling is also implicated in a variety of cancers [138, 139]. Wnt/β-catenin pathway is expressed in the adult lung epithelium and overexpressed during inappropriate EMT in IPF [116, 140]. Although intensive efforts have been made, the Wnt signaling pathway remains difficult to target. Tight cross-talk among MET and Wnt signaling is known in tissue morphogenesis as well as in several cancer types [141–144]. Conversely, an intricate interaction between TGFβ and MET signaling is well discovered. The TGFβ superfamily is known to play a critical role in the regulation of cell differentiation and proliferation. The TGF cascade mainly involves the activation of the cytoplasmic signaling molecules Smad2 and Smad3 for the TGF/activin pathway and Smad1/5/5/9 for the TGF/bone morphogenetic protein (BMP) pathway [145–149]. In particular the cross talk between TGFβ/BMP pathways is implicated in several biological programs and involved in a number of progressive disease, among which cancer [150–153]. The role of the TGFβ pathway has been extensively studied in IPF. Overexpression of its effects is induced by persistent injury and is, in turn, associated to the aberrant lung remodeling and fibrogenesis by activating myofibroblasts to produce extracellular matrix [130, 154]. Many drugs have been developed to target the TGFβ family signaling cascade [155]. In particular the FDA approved in 2014 the TGFβ1 inhibitor Pirfenidone for IPF therapy [156–159]. HGF can antagonize the TGFβ profibrotic phenotypes by several mechanisms, mainly by transcriptional TGFβ down-modulation [160] and ERK-mediated inhibition of Smad proteins [161, 162]. Nevertheless, more recently it has been shown that overexpression of the HGF receptor MET together with CD44 isoform 6 (CD44v6) sustains the TGFβ signaling in IPF through an autocrine loop [27]. Another relevant issue is that there is considerable experimental evidence that tissue hypoxia is associated with the onset of fibrosis. However, although chronic hypoxemia can clinically characterize IPF, the role of local hypoxia as a driver of the progressive fibrotic nature of the disease has not been fully clarified. Low oxygen tension has variable effects on cellular proliferation depending on the cell type. While arresting alveolar epithelial cell proliferation, low oxygen tension has been shown to promote normal fibroblast proliferation, leading to the possibility that hypoxia may promote IPF fibroblasts proliferation [163–166]. It has been recently reported that a pathological feed-forward loop may exist in the IPF lung, in which hypoxia promotes IPF fibroblasts proliferation via stimulation of miR-210 expression, which in turn worsens hypoxia [167]. More importantly, molecular links are beginning to emerge between hypoxia, EMT and stemness. During embryogenesis hypoxia contributes to the induction of niches that maintain pluripotent cells. During carcinogenesis, hypoxia has the potential to exert significant effect on the maintenance and evolution of cancer stem cells. Moreover solid tumor hypoxia is a well-known factor in tumor aggressiveness and invasive potential [168]. It is plausible that hypoxia-induced MET up-regulation may occur in IPF as well, and can cooperate in triggering the regenerative/reparative processes that define the disease onset and progression. This hypothesis questions the use of anti-angiogenic agents in IPF, as in cancer therapy; deprivation of a blood supply, and thus of oxygen could in fact induce, besides the desirable tissue necrosis, a dangerous “invasive switch”. It would therefore be advisable to combine anti-angiogenic treatments (e.g. nintedanib) with an anti-MET agent to prevent these potential drawbacks. Regarding the rationale of MET therapeutic blockade in IPF, another key point must be underlined. Since IPF is, by definition, polyclonal, the reported MET activation may be independent of on the phenomena of oncogenic addiction associated with structural alterations related to cancer clonal evolution or—considering the role played by HGF—to ligand–receptor autocrine circuits, which frees cells from the need for a paracrine supply of growth factor. In this perspective, cells undergoing EMT often take advantage of the physiological function of MET as an “expedience” [169] to gain a selective advantage in IPF progression.

## Conclusion

The molecular pathways involved in the metastatic process in cancer are shared with IPF. Among them the MET-driven invasive growth program plays a crucial role. As discussed above, MET activation governs a number of physiological and pathological processes that modulate dynamic changes and plasticity of tissues. If in cancer MET activation enables cells to overcome damage induced by targeted agents and ionizing radiation, there is enough evidence to sustain that in IPF the versatility of the MET-mediated biological responses may promote tissue remodeling by integrating growth, survival and migration cues in response to abnormal environmental stimuli or cell-autonomous perturbations in absence of addiction phenomena. More likely, MET expression in myofibroblasts behaves as in cancer stem cells, where it sustains the inherent self-renewing, self-preserving and invasive growth phenotype [8, 19]. These notions indicate three clinical implications: (1) MET is a versatile candidate for targeted therapeutic intervention in IPF. (2) Targeted therapies against MET could be effective as a combinatorial approach to restrict disease progression, rather than being used as single front-line approaches. (3) Validation of MET expression as a biomarker is mandatory to developing therapies for IPF based on MET inhibition.

## Abbreviations

IPF: idiopathic pulmonary fibrosis
FF: fibroblast foci
RTK: receptor tyrosine kinases
EMT: epithelial-mesenchymal transition
HGF: hepatocyte growth factor
ECM: extracellular matrix
IGF: insulin-like growth factor
NGF: nerve growth factor
EGF: epidermal growth factor
MMP: metalloproteinases
