# Erratum to: Ockham’s razor for the MET-driven invasive growth linking idiopathic pulmonary fibrosis and cancer

**DOI:** 10.1186/s12967-017-1295-4

**Published:** 2017-09-19

**Authors:** Giulia M. Stella, Alessandra Gentile, Alice Balderacchi, Federica Meloni, Melissa Milan, Silvia Benvenuti

**Affiliations:** 1 Pneumology Unit, Cardiothoracic and Vascular Department, IRCCS Policlinico San Matteo Foundation and University of Pavia Medical School, Piazzale Golgi 19, 27100 Pavia, Italy; 2 Investigational Clinical Oncology (INCO), IRCCS Candiolo Cancer Institute-FPO, Candiolo, 20060 Turin, Italy; 3 Experimental Clinical Molecular Oncology (ECMO), IRCCS Candiolo Cancer Institute-FPO, Candiolo, 20060 Turin, Italy

## Erratum to: J Transl Med (2016) 14:256 DOI 10.1186/s12967-016-1008-4

In the original version of this article [1], published on 2 September 2016, the name of author ‘Alice Balderacchi’ was wrongly displayed. In this Erratum the incorrect name and correct name are shown. The original publication of this article has been corrected.

Originally the author name has been published as:

- Alice Baderacchi

The correct author name is:

- Alice Balderacchi
